# Incidence of Apoptosis in the Lymphoid Organs of Normal or Malaria Infected Mice is Decreased in CD18 and Urokinase - Receptor (UPAR, CD87) Deficient Mice

**DOI:** 10.1155/2001/75259

**Published:** 2001

**Authors:** Pierre Francois Piguet, Chen da Laperrousaz, Christian Vesin, Yves Donati

**Affiliations:** Department of Pathology University of Geneva 1 rue M. Servet CMU CH 1211 Switzerland

**Keywords:** malaria, CD18, CD87, apoptosis, IgG, lymphocyte

## Abstract

Incidence of apoptosis was investigated in the spleen and lymph nodes of +/+, CD18 -/- and urokinase receptor (uPAR, CD87) -/- mice, untreated or *Plasmodium Berghei Anka* (PbA) infected. In non infected mice, incidence of apoptosis was lower in the lymph nodes of CD18 -/- and uPAR -/- than in +/+ mice, as seen by FACS analysis to count the number of hypodiploid and Annexin-V binding cells. Infection of mice with PbA resulted in a marked increase in the size of spleen and lymph nodes 7–8 days after infection, which was slightly higher in uPAR -/- and CD18 -/- than in +/+ mice. PbA infection increased about 7 fold the incidence of apoptosis in the lymphoid organs of +/+, especially in the white pulp and germinal centers of the spleen and lymph nodes, while in contrast it was unchanged in PbA infected CD18 -/- or uPAR -/- mice. Serum IgG levels, and number of circulating leukocytes were significantly higher in both uPAR and CD18 -/- than in +/+ mice. These results indicate that the CD18 and uPAR surface molecules, which are known to be associated in the cell membrane, have an important influence upon the incidence of cell survival in both normal or stimulated lymphoid organs.


*Developmental Immunology*, 2001, Vol. 8(3-4), pp. 183-191
Reprints available directly from the publisher
Photocopying permitted by license only

© 2001 OPA (Overseas Publishers Association)
N.V. Published by license under
the Harwood Academic Publishers imprint,
part of The Gordon and Breach Publishing Group,
a member of the Taylor and Francis Group

### Incidence of Apoptosis in the Lymphoid Organs of Normal or Malaria Infected Mice is Decreased in CD18 and Urokinase - Receptor (UPAR, CD87) Deficient Mice

PIERRE FRANCOIS PIGUET*, CHEN DA LAPERROUSAZ, CHRISTIAN VESIN and YVES DONATI

*Department of Pathology, University of Geneva, 1 rue M. Servet, CMU, CH 1211, Switzerland*

Incidence of apoptosis was investigated in the spleen and lymph nodes of +/+, CD18 -/- and urokinase receptor (uPAR, CD87) -/- mice, untreated or *Plasmodium Berghei Anka* (PbA) infected. In non infected mice, incidence of apoptosis was lower in the lymph nodes of CD18 -/- and uPAR -/- than in +/+ mice, as seen by FACS analysis to count the number of hypodiploid and Annexin-V binding cells. Infection of mice with PbA resulted in a marked increase in the size of spleen and lymph nodes 7–8 days after infection, which was slightly higher in uPAR -/- and CD18 -/- than in +/+ mice. PbA infection increased about 7 fold the incidence of apoptosis in the lymphoid organs of +/+, especially in the white pulp and germinal centers of the spleen and lymph nodes, while in contrast it was unchanged in PbA infected CD18 -/- or uPAR -/- mice. Serum IgG levels, and number of circulating leukocytes were significantly higher in both uPAR and CD18 -/- than in +/+ mice. These results indicate that the CD18 and uPAR surface molecules, which are known to be associated in the cell membrane, have an important influence upon the incidence of cell survival in both normal or stimulated lymphoid organs.

*Keywords:* malaria, CD18, CD87, apoptosis, IgG, lymphocyte

### INTRODUCTION

In mice, infection by *Plasmodium* bergei *ANKA* (PbA) results in a marked increase in the size of the lymphoid organs (Freeman and Parish, 1978). The increase in size might be the result of both a polyclonal activation, involving T and B lymphocytes (Freeman and Parish, 1978), and a specific anti-malaria response, reviewed in (Good and Doolan, 1999).

Tissue homeostasis is regulated in great part by cell proliferation and cell death, which takes the form of programmed cell death (PCD) or apoptosis (Nagata, 1997). For mobile cells as those of the lymphoid organs, emigration is an additional possibility to regulate the cell capital. Some cell surface molecules, such as TNFR1 (CD120a), Fas (CD 95), and CD40 (Nagata, 1997), are known to directly trigger the apoptotic pathway in various cell types, including lymphocytes. IL-2 and its receptor are also suspected to be involved in the regulation of survival of lymphoid cells since their absence result in lympho-adenopathy and auto-immunity, reviewed in (Refaeli et al. 1999).

During the study of the role of adhesion molecules in the mortality of mouse severe (or cerebral) malaria,

* Corresponding author.

we unexpectedly observed that the incidence of apoptosis seen within the lymphoid organ was markedly decreased in urokinase-receptor (uPAR, CD87) and CD18 (β2 integrin) deficient mice. Recent evidence indicates that the β2 integrin (CD18-CD11 heterodimer) is associated within the cell membrane with the urokinase receptor (uPAR, CD87, reviewed in (Chapman, 1997)). The uPAR is a 55 KD glycosyl-phosphatidylinositol (GPI)- linked surface receptor known to bind urokinase plasminogen activator (uPA), plasminogen activator inhibitor 1 (PAI-1) and vitronectin. Binding of uPA or PAI-1 to uPAR is believed to influence the conformation of the uPAR and its interaction with other integrins and thus the function of the integrin. The traffic of PMNs in the mouse peritoneum has been shown to be delayed in uPAR -/- mice (Wei et al. 1996). In human, CD18 deficiency is known to results in a leukocyte adhesion deficiency syndrome (Kishimoto et al. 1989). In mice, an hypomorphic deletion of CD18 or a more complete deletion of CD18 have been reported to result in leukocytes adhesion impairement, leukocytosis in the blood, and hypergammaglobulinemia (Wilson et al. 1993; Scharffetter-Kochanek et al. 1998).

In the present report we investigated the incidence of apoptosis in the lymphoid organs of normal or PbA infected mice, using various methods. Apoptosis was markedly and similarly decreased in either CD18 or uPAR deficient mice, indicating that CD18 and uPAR molecules are implicated not only in adhesion but also in the regulation of lymphocyte life span in vivo.

### METHODS

### Mice

C57BL/6J, (+/+) and C57BL/6J-Itgb2 (CD18-/-) (Wilson et al. 1993), were purchased from Jackson Laboratories, Bar Harbor, Maine. uPAR -/- genetically deficient mice, isolated on the C57BL/6 × 129 background (Bugge et al. 1995; Dewerchin et al. 1996), were obtained from P. Carmeliet, Belgium and bred in our animal facility. Mice of either sex were used between 2–5 months of age. C57BU6 were used as +/+ controls of the CD18 -/-, while C57BL/6x129 Fl were used as +/+ controls of the uPAR-/- mice.

### Plasmodium Berghei Anka infection (PbA)

PbA has been passaged in rodents for decades (Wright, 1968). Mice were injected iv with $10^4$ parasitized red blood cells (pRBC).

### Blood Formulation

Blood (0.05 ml) was isolated from the retro-orbital plexus using heparinized capillaries and diluted in EDTA (1 % final). Blood elements were counted in a cell counter (Casy 1, Schärfe system, D-72760 Reutlingen).

### Serum Ig levels

Concentrations of serum IgM and IgG were determined by ELISA, as described (Luzuy et al. 1986).

### Cell preparation

Spleen cells were prepared by teasing a fragment of the spleen with forceps. Lymph node cells (LNC) were isolated from the iliac and axillary nodes. Lymph nodes (LN) were disrupted by teasing with a tissue homogeneizer, as described elsewhere (Piguet et al. 1975).

### Fluorescent cell sorter analysis (FACS)

Cells isolated from the spleen or the peripheral lymph nodes (LNC) were stained with Propidium Iodide to determine their DNA content (Darzynkiewicz et al. 1992). Binding of Annexin-V was determined as described elsewhere (Martin et al. 1995).

Cells were analyzed in a FACScan Flow Cytometer (Becton Dickinson, San Jose, CA, USA). The events corresponding to leukocytes were identified in SSC and FSC in a logarithmic acquisition mode and gated. The data from $10^4$ cells were collected using the Lysis II software.

### Assay for DNA strand break in situ

TUNEL (terminal transferase dUTP nick end-labeling) assay was performed on formalin-fixed tissue sections (5 μm) as described previously (Gavrieli et al. 1992). Reagents were purchased from Boehringer Mannheim (Mannheim, Germany). Briefly, tissue sections were mounted onto slides pretreated with 3-aminopropylethoxysilane (Merck AG, 8953-Switzerland), baked over night at 55°C, dewaxed and rehydrated. To facilitate access of the reagent to DNA fragments, slides were treated with 30 μg/ml proteinase K for 15 min and also incubated in the microwave for 1 min in citrate buffer 0.01M, pH 6.0. Subsequent end-labeling with terminaldeoxynucleotyl transferase (TdT) (0.3U/ml) in TdT buffer containing 2 mM/l biotin 16-UTP was carried out for 1 hour at 37°C. Sections were incubated with strepatavidin-biotin-horseradish-peroxidase complex (Dako, 6302-CH) and stained with di-aminobenzidine.

### DNA internucleosomal degradation (laddering)

This was performed as described elsewhere (Mantell et al. 1997). In brief, organs were homogenized by polytronic disruption in PBS 10mM EDTA (10mg of tissue in 0.5 ml). The homogenate was then centrifuged at 13000 rpm for 20 min at 4°C. The supernatant, enriched in DNA molecules of small size was kept and treated for 30 min at 37°C with 20μg/ml RNase A and another 30min with 200 μg/ml proteinase K. After 3 phenol/chloroform extractions, 1/10 sodium acetate 3M, pH 4.8, was added and DNA was precipitated with 1 volume of isopropanol. The pellet was centrifuged at 13000 rpm for 10 min at 4°C, washed with 70% ethanol, dried and resuspended in 10mM Tris, 1mM EDTA, pH 8.0. The samples were run in 1% agarose gel and DNA fragmentation revealed with ethidium bromide.

### Statistical evaluation

Values from different groups of measurements were compared with the Student t-test or the non parametric Mann & Whitney test (Mann and Whitney, 1947).

### RESULTS

### 1) Effect of CD18 and uPAR deficiency on the size of the lymphoid organs in normal or PbA infected mice

PbA infection results in an important increase in the size of the lymphoid organs. In LN, the increase in cellularity was of similar magnitude in +/+ and uPAR -/-, while in was significantly more pronounced in CD18-/- than in their +/+ controls (Fig 1). In spleen which is both a lymphoid and hematopoietic organ, the increase in size was about 3 fold 8th days after infection (Fig 1) and significantly more important in uPAR -/- than in their +/+ controls (Fig 1).

### 2) Effect of CD18 and uPAR deficiency on the incidence of apoptosis in the lymphoid organs of non treated or PbA infected mice

### *A) Histology*

In the white pulp of the lymphoid organs of untreated mice, apoptotic cells were rarely observed on Hematoxilin-Eosin stained sections (Fig 2A). Incidence of apoptosis was markedly increased by PbA infection, especially in the white pulp of the spleen (Fig 2B) and the germinal centers of the LN. Apoptosis frequently occurred in clusters (Fig 2) and it is likely that these pycnotic cells are already within macrophages. In CD18 -/- or uPAR -/- mice, PbA infection did not increase significantly the number of apoptotic cells in the white pulp (Fig 2 C&D).

### *B) DNA strand breaks (tunel)*

PbA infection increased the number of TdT labeled cells in the white pulp from +/+ mice, while in the red pulp, changes were less striking with apoptotic cells being present in both control and infected +/+ mice (2E). In uPAR -/- mice, no increase in the incidence of TdT labeled cells was observed (Fig 2 F).

### *C) DNA laddering*

Internucleosomal DNA degradation is a characteristic of apoptosis. DNA laderring was detectable in the

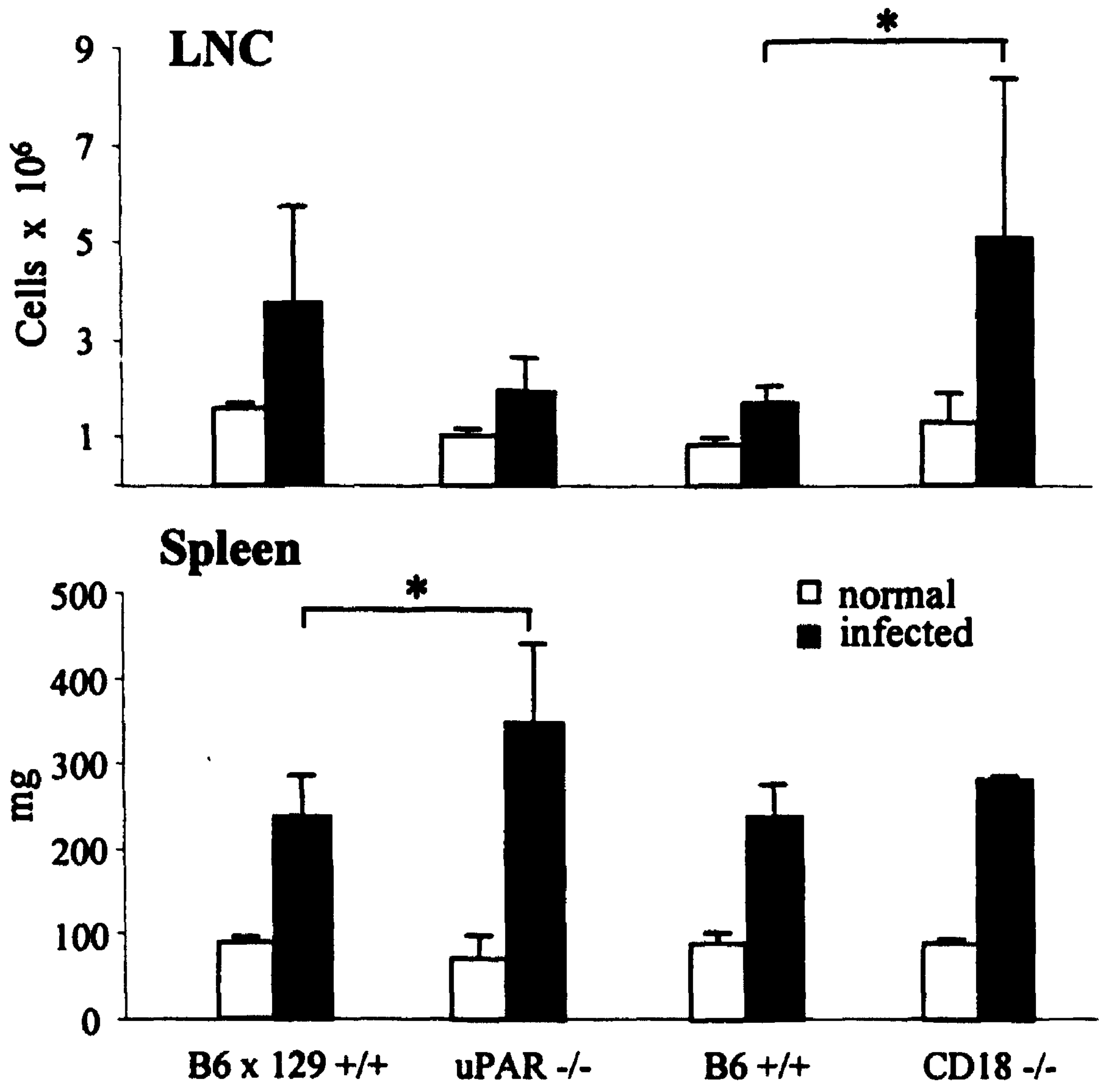

FIGURE 1 Effect of PbA infection on the size of the lymphoid organs in +/+, uPAR-/- and CD18 -/- mice Top, number of cells within one axillary lymph node. Bottom, size of the spleen, in mg Results are the mean of the values observed with 5 mice. *p<0.05

spleen of normal mice in contrast to other organs such as the brain where it is undetectable (Fig 3). Importance of DNA laddering in the spleen was increased by PbA infection of +/+ mice (Fig 3). In infected uPAR-/- and CD18 -/- mice, laddering was somewhat decreased compared to the +/+ infected controls, but with important individual variations within the groups (not shown).

### *D) Annexin-V binding detected with the FACS*

Annexin-V binding is considered to be an early change in the membrane of cells engaged in the apop-

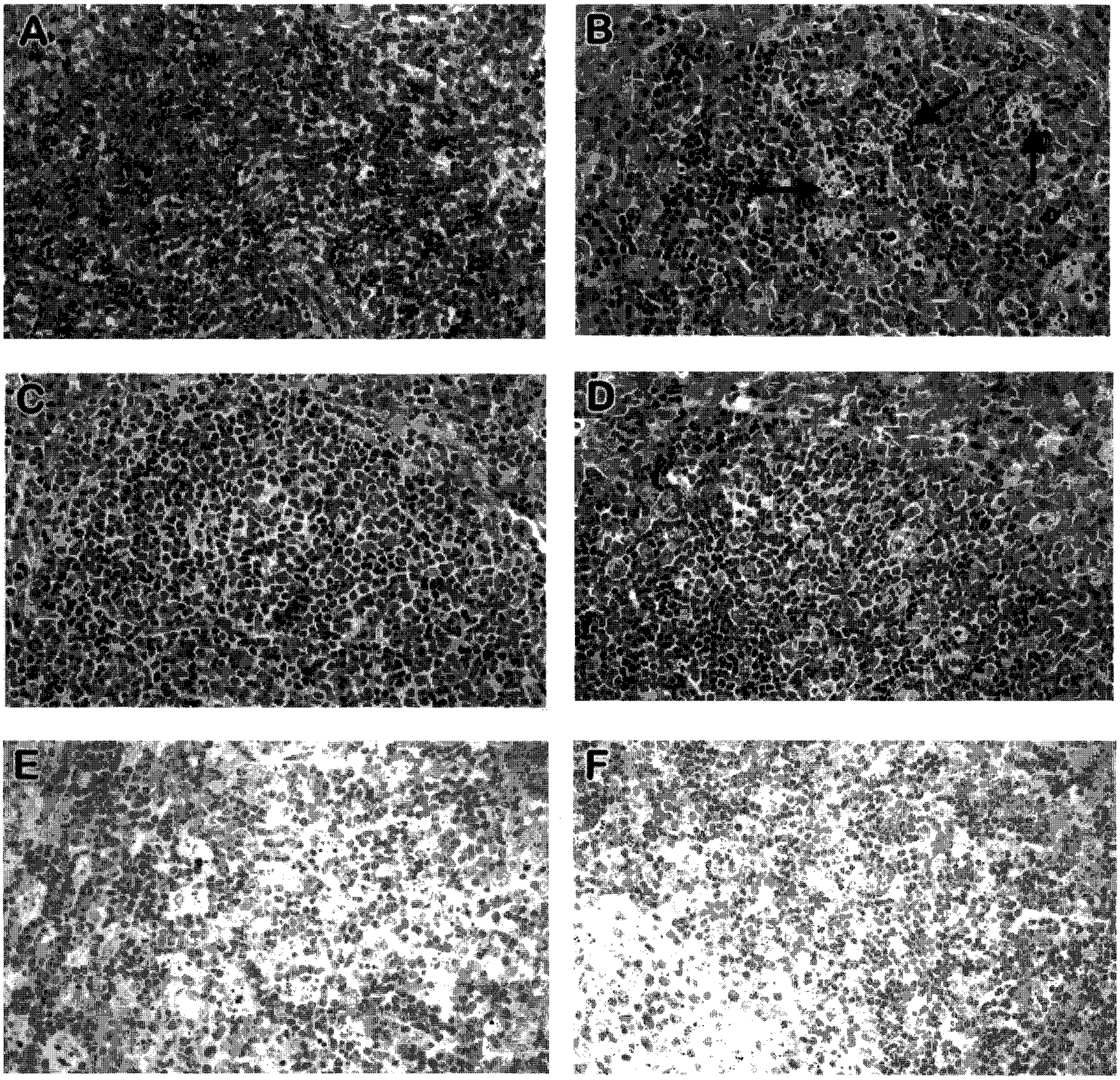

FIGURE 2 White pulp of control or PbA infected +/+, CD18 -/- and uPAR -/- mice. Splenic white pulp of a non infected +/+ (A), and PbA infected, +/+ (B), uPAR-/- (C), and CD18 -/- mice (D). Numerous foci of pycnotic nuclei are visible in B (arrows). TdT end labeling of the splenic white pulp of PbA infected +/+ (E), or uPAR -/- (F). Numerous labeled cells (red) are visible in E. Magnification; 400x totic pathway (Martin et al. 1995). In order to focalize the study on lymphoid cells, we examined dissociated LNC. In non infected mice, the % of Annexin-V-binding cells was of the order of 5–10% in +/+ mice and significantly less in CD18 -/- or in uPAR -/- mice (Fig 4). In PbA infected mice, the % of Annexin binding was increased by about 7 fold in +/+, but neither in CD18 -/- nor in uPAR-/- (Fig 4).

***E) Hypodiploid cells detected by PI labeling***

Cells in the apoptotic pathway become hypodiploid when they are in an advanced stage of PCD. In gen-

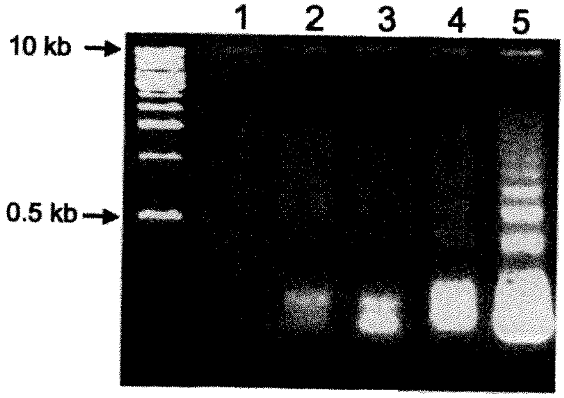

FIGURE 3 DNA laddering DNA internucleosomal degradation of DNA seen in a normal brain (lane 1) or spleen from a +/+ (lane 2 & 3) and PbA infected mice (4 & 5)

eral, the incidence of hypodiploid cells was much lower than that of Annexin-V binding i.e. ranging between 0.5–1 and 5–10% for normal or PbA infected LNC respectively (Fig 5). In non infected mice, we detected no significant difference in the % of hypodiploid cells, seen in the M5 window of Fig 5, between +/+, CD18 -/- and uPAR -/- mice (Fig 5). In PbA infected mice, the % of hypodiploid cells was significantly increased in +/+ (about 4 fold), but not in CD18-/- or in PAR -/- mice (Fig 5).

### 3) Effect of uPAR deficiency on blood leukocyte counts and serum Ig levels

The counts of circulating leukocytes were significantly higher in uPAR -/- than in +/+ mice, respectively of 8 (2) and 5 (1) × $10^3$ cells/μl.

Serum levels of various IgGs are known to be elevated in CD18-/- mice (Scharffetter-Kochanek et al. 1998). In uPAR-/-, the serum IgG levels were also higher in uPAR-/- than in +/+, with titers of 1200 (200) and 600 (50) μg/ml respectively at 3 months of age.

We found increase in leukocytes counts and serum IgG levels in CD18-/- mice (not shown), in accord to what has been reported (Scharffetter-Kochanek et al. 1998), similar to those seen in uPAR -/- mice.

### DISCUSSION

Size of the lymphoid organs is the result of a balance between cell proliferation, cell death and possibly also emigration. Apoptosis or PCD is believed to be an essential mechanism of regulation of tissue homeostasis and, in case of the lymphoid organs, in the number of lymphocytes available as effectors of the immune response. Two types of apoptosis in lymphoid cells have been distinguished, one named passive cell death, elicited by the withdrawal of nutrient or growth factor, and the other activation-induced cell death (Refaeli et al. 1999). In the present study, we observed a markedly increased incidence of apoptosis in the malaria stimulated lymphoid organs of +/+, but not of uPAR-/- or CD18 -/- mice, indicating an important increase of apoptosis during immunostimulation and also an important role of these receptors in the activation associated apoptosis. However, in non infected mice, in which the quantitative evaluation of apoptosis is less precise due to its low incidence, we did also detect a similar trend, with a significantly lower incidence of Annexin-V binding cells in the lymphoid cells of uPAR and CD18 deficient mice than in their +/+ controls (Fig 4). This was not see in the counts of hypodiploid cells, perhaps because a majority of Annexin-V + cells are subsequently taken up by macrophages (as suggested by the histology, Fig 1B) and cannot be seen as isolated hypodiploid cells. The present results suggest therefore that the role of uPAR and CD18 in the regulation of apoptosis is similar in lymphoid cells from conventional mice (i.e. stimulated by the "normal" environment) and those massively stimulated by PbA infection.

Lymphocyte apoptosis has been mainly attributed to the Fas, the CD40 and the TNF receptors, which are known to activate the PCD in lymphocytes, reviewed in (Beutler and van Huffel, 1994). Regarding CD18, a recent observation demonstrates that the incidence of apoptosis in polymorphonuclear leukocytes of a peritoneal exudate is markedly decreased in CD18/CD11b -/-, compared to +/+ mice (Wang and Leonardo, 1997), an observation similar to the present one concerning lymphocytes. CD18 and uPAR are not known to convey death signals. However it is possible

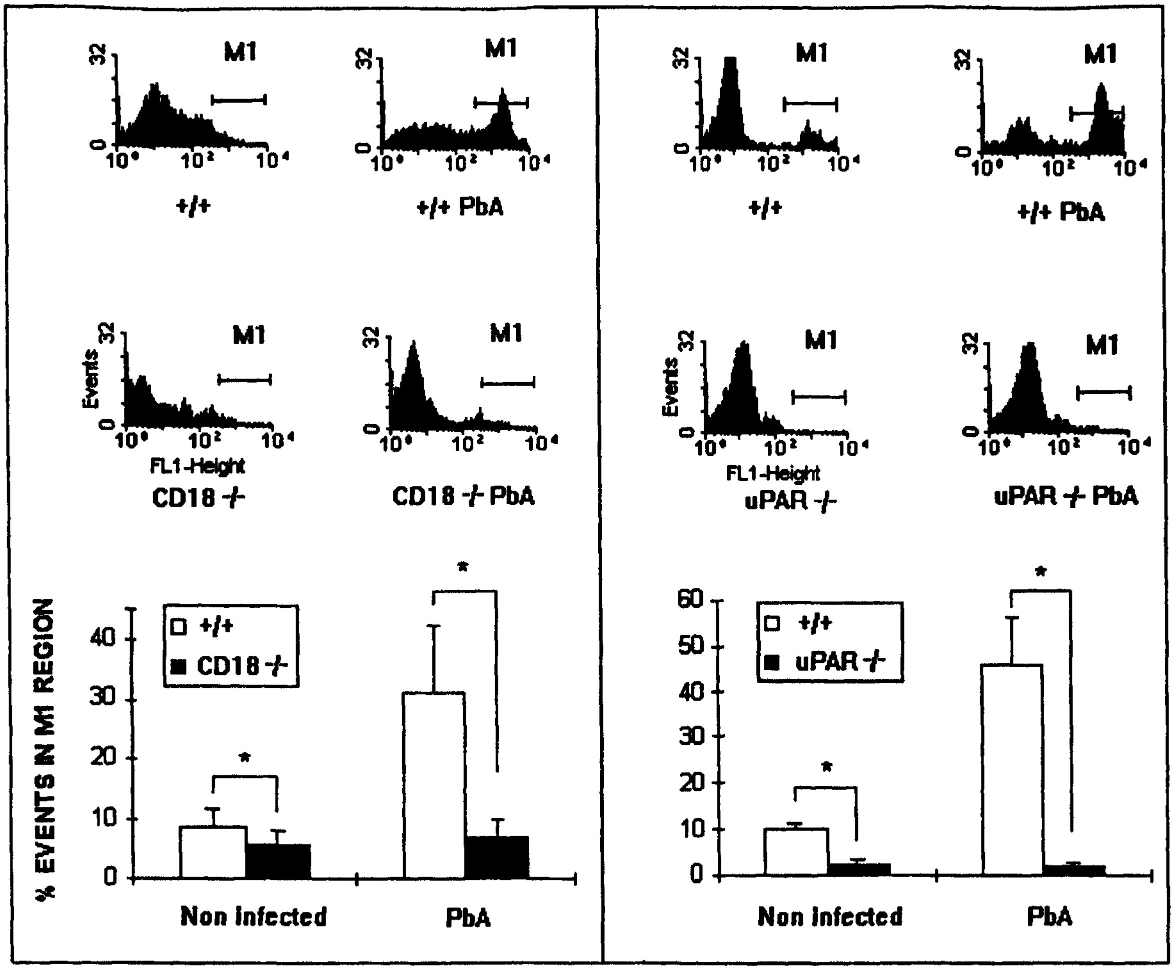

FIGURE 4 Incidence of Annexin-V binding cells detected by FACS in the LNC of +/+, CD18-/-, and uPAR-/-, untreated or PbA infected mice Top: representative examples of the fluorescence intensity, seen in ordinate on a logarithmic scale. Annexin-V + cells are present in the M1 gate. Bottom, (bar graph) mean (sd) of the percentage of cells present in the M1 gate seen in five individual mice. LNC were isolated on the 8th day after PbA infection. *$p < 0.05$

that they modulate the incidence of PCD in activated cells by their effect on stretch, since for epithelial cells, there is evidence that lack of anchorage might promote apoptosis (Ruoslahti, 1997). Alternatively, β2 integrin might be critical in some macrophages-lymphocytes, or T lymphocyte-B lymphocytes interactions involved in apoptosis, as suggested by in vitro studies (Gladstone et al. 1996; Wang and Leonardo, 1997). uPAR and CD18 are known to be associated in the cell membrane (Chapman, 1997); thus the similar decrease of the incidence of apoptosis induced by the deletion of one or the other is therefore consistent with the possibility that these receptors are also functionally associated.

A decrease in apoptosis is expected to result in an increase in the size of lymphoid organs, what is observed in FAS, CD40, but not with TNFR deficient mice (Refaeli et al. 1999). CD18 deficiency is known to elicit a leukocytosis with little gross alterations of the lymphoid organs. Leukocytosis is generally attrib-

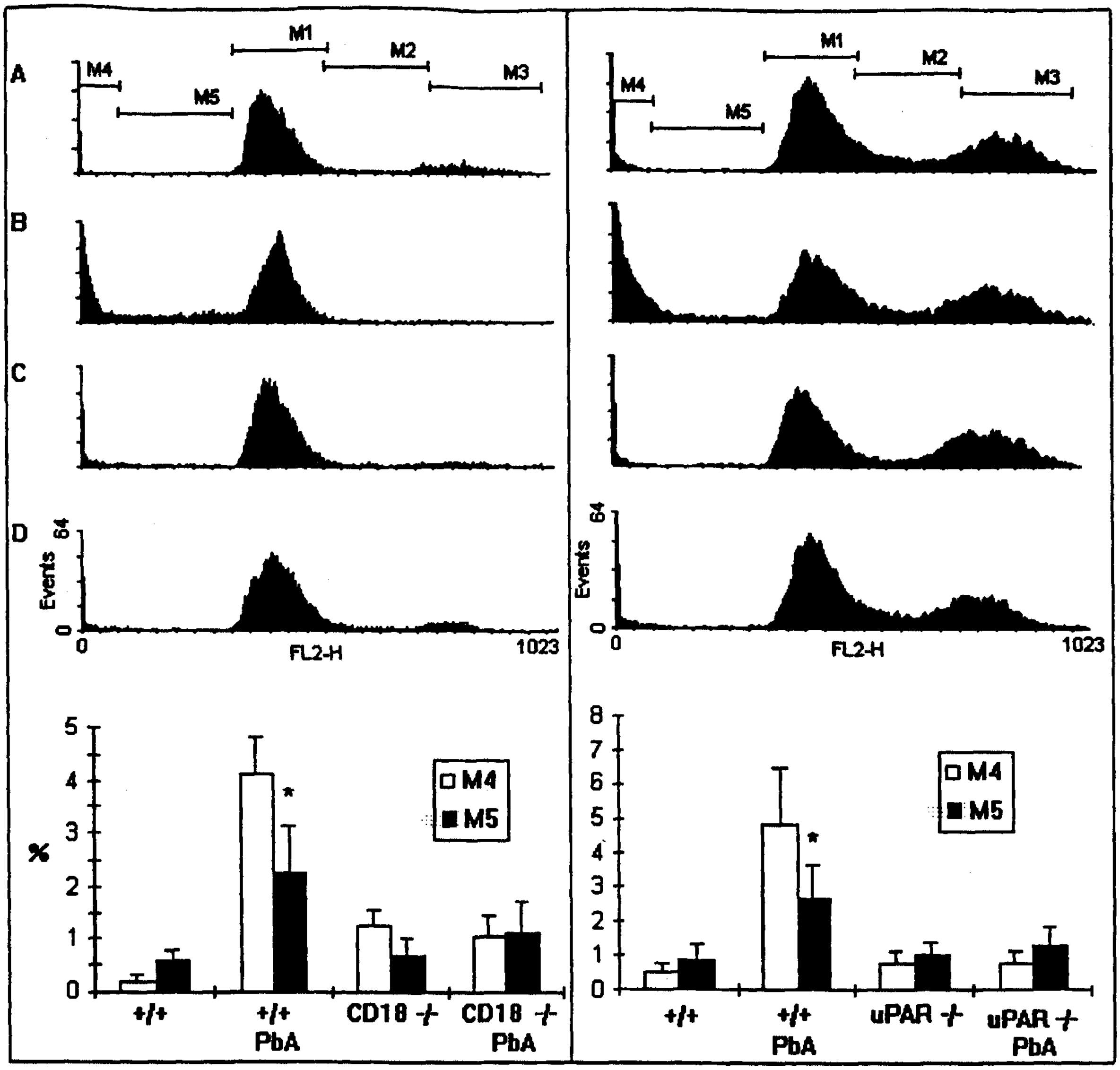

FIGURE 5 Incidence of hypodiploid cells detected by FACS in LNC from +/+, CD18-/- or uPAR -/-, untreated or PbA infected mice Top, A-D are results from a representative experiment: +/+ (A); +/+; PbA (B); -/-, (C); -/- PbA (D). The windows correspond to M1=2N; M2= S phase; M3=4N; M5=hypodiploid, M4=necrotic cells. Bottom, the results are a mean (sd) of the % of events in the indicated window in individual mice (n=4). LNC were collected on the 8th day after PbA infection and the DNA content measured by PI labeling. *$p<0.05$ compared to +/+ non infected uted to a defect in emigration from the blood compartment but since it is not associated with a depletion of the lymphoid organs (rather the reverse) it is very likely that leukocytosis is only one element of a generalized increase of the pool of lymphoid cells due to a deficiency of apoptosis. CD18 deficiency is also known to increase the serum IgG and IgM levels (Scharffetter-Kochanek et al. 1998), alterations which might be related to an increased life span of plasma cells. Here also, for what concern the Ig levels or the size of the lymphoid cell pool, uPAR deficiency had very similar consequences to those of CD18 deficiency. This therefore also strongly support the close functional association of these receptors, as proposed by (Chapman, 1997).

Mortality during the acute phase of PbA infection, due to "severe or cerebral malaria" is known to be immunodependant and to require T lymphocytes (Grau et al. 1986). CD18 deficiency is not known to have a major influence on the immune response in vivo (Wilson et al. 1993) but it is seems that a more detailed study of the immune response of both uPAR and CD18 deficient mice remains to be done. The present observations raises nevertheless the question whether the incidence of apoptosis in the lymphoid organs influence the severity of malaria. This appear not to be the case however since the acute mortality of PbA infection was decreased in uPAR -/-, but not in CD18 -/- mice (unpublished observations).

### *Acknowledgements*

This work was supported by a grant no 32 47284.96 from the Swiss national science foundation. We are grateful to D. Wohlwend in the Centre de Fluorographie du Centre Medical Universitaire for his help in the FACS analysis.

### *References*

Beutler B, van Huffel C. (1994). Unraveling function in the TNF ligand and receptor families. Science **264**: 667–8.

Bugge TH, Suh TT, Flick MJ, Daugherty CC, Romer J, Solberg H, Ellis V, Dano K, Degen JL. (1995). The receptor for urokinase-type plasminogen activator is not essential for mouse development or fertility. J Biol Chem **270**: 16886–94.

Chapman HA. (1997). Plasminogen activators, integrins and the coordinated regulation of cell adhesion and migration. Current Opinion in Cell Biology **9**: 714–24.

Darzynkiewicz Z, Bruno S, Del Bino G, Gorczyca W, Hotz MA, Lassota P, Traganos F. (1992). Features of apoptotic cells measured by flow cytometry. Cytometry **13**: 795–808.

Dewerchin M, Van Nuffelen A, Wallays G, Bouché A, Moons L, Carmeliet P. (1996). Generation and characterization of urokinase receptor-deficient mice. J Clin Invest **97**: 870–8.

Freeman RR, Parish CR. (1978). Polyclonal B-cell activation during rodent malarial infections. Clin Exp Immunol **32**: 41–5.

Gavrieli Y, Sherman Y, Ben-Sasson SA. (1992). Identification of programmed cell death in situ via specific labelling of nuclear DNA fragmentation. J Cell Biol **119**: 493–501.

Gladstone RC, Nielsen P, Borregaard N, Ledbetter JA, Svejgaard A, Odum N. (1996). Apoptosis following interleukin-2 withdrawal from T cells: evidence for a regulatory role of CD18 (beta-2 intergrin) moelcules. Tissue Antigens **48**: 127–35.

Good MF, Doolan DL. (1999). Immune effector mechanisms in malaria. Curr Opin Immunol **11**: 412–9.

Grau GE, Piguet PF, Engers HD, Louis JA, Vassalli P, Lambert PH. (1986). L3T4+ T lymphocytes play a major role in the pathogenesis of murine cerebral malaria. J Immunol **137**: 2348–54.

Kishimoto TK, Larson RS, Corbi AL, Dustin ML, Staunton DE, Springer TA. (1989). The leukocyte integrins. Adv Immunol **46**: 149–82.

Luzuy S, Merino J, Engers H, Izui S, Lambert PH. (1986). Autoimmunity after induction of neonatal tolerance to alloantigens: role of B cell chimerism and F1 donor B cell activation. J Immunol **136**: 4420–6.

Mann HB, Whitney DR. (1947). On a test of whether one or two random variables is stochastically larger than the other. Ann Math Statist **18**: 50–60.

Mantell L, Kazzaz J, Xu J, Palaia T, Piedboeuf B, Hall S, Rhodes GC, Niu G, Fein AM, Horowitz S. (1997). Unscheduled apoptosis during acute lung injury. Cell Death and Differentiation **4**: 600.

Martin SJ, Reutelinsperger CPM, McGahon AJ, Rader JA, van Schie RCAA, LaFace DM, Green DR. (1995). Early redistribution of plasma membrane phosphatidylserine is a general feature of apoptosis regardless of the initiating stimulus: inhibiton by overexpresion of Bcl-2 and Abl. J Exp Med **182**: 1545–56. Nagata S. (1997). Apoptosis by death factor. Cell **88**: 355–65.

Piguet PF, Dewey HK, Vassalli P. (1975). Study of cells proliferating in parent versus F1 hybrid mixed lymphocyte culture. J Exp Med **141**: 775.

Refaeli Y, Van Parijs L, Abbas AK. (1999). Genetic models of abnormal apoptosis in lymphocytes. Immunolgical Reviews **169**: 273–82.

Ruoslahti E. (1997). Stretching is good for a cell. Science **276**: 1345–6.

Scharffetter-Kochanek K, Lu H, Norman K, van Nood N, Munoz F, Grabbe S, McArthur M, Lorenzo I, Kaplan S, Ley K, et al. (1998). Spontaneous skin ulceration and defective T cell function in CD18 null mice. J Exp Med **188**: 119–31.

Wang J, Leonardo MJ. (1997). Essential lymphocyte function associated 1 (LFA-1): intercellular adhesion molecule interactions for T cell-mediated B cell apoptosis by FAS/APO-1/CD95. J Exp Med **186**: 1171–6.

Wei Y, Lukashev M, Simon DI, Bodary SC, Rosenberg S, Doyle MV, Chapman HA. (1996). Regulation of integrin function by the urokinase receptor. Science **273**: 1551–5.

Wilson RW, Ballantyne CM, Smith CW, Montgomery C, Bradley A, O'Brien WE, Beaudet AL. (1993). Gene targetting yields a CD18 mutant mouse for study of inflammation. J Immunol **151**: 1571–8.

Wright DH. (1968). The effect of neonatal thymectomy on the survival of golden hamsters infected with Plasmodium berghei. Br J Exp Pathol **49**: 379–84.

